# Impact of Consolidative Unrelated Cord Blood Transplantation on Clinical Outcomes of Patients With Relapsed/Refractory Acute B Lymphoblastic Leukemia Entering Remission Following CD19 Chimeric Antigen Receptor T Cells

**DOI:** 10.3389/fimmu.2022.879030

**Published:** 2022-04-26

**Authors:** Qianwen Xu, Lei Xue, Furun An, Hui Xu, Li Wang, Liangquan Geng, Xuhan Zhang, Kaidi Song, Wen Yao, Xiang Wan, Juan Tong, Huilan Liu, Xin Liu, Xiaoyu Zhu, Zhimin Zhai, Zimin Sun, Xingbing Wang

**Affiliations:** ^1^ Department of Hematology, The First Affiliated Hospital of University of Science and Technology of China (USCT) (Anhui Provincial Hospital), Division of Life Sciences and Medicine, University of Science and Technology of China, Hefei, China; ^2^ Hematology Department, The Second Hospital of Anhui Medical University (SHAMU), Hefei, China

**Keywords:** chimeric antigen receptor (CAR), unrelated cord blood transplantation, acute B lymphoblastic leukemia, CD19, prognosis

## Abstract

**Background:**

While chimeric antigen receptor (CAR)-T cell therapy is becoming widely used in hematological malignancies with remarkable remission rate, their high recurrence remains an obstacle to overcome. The role of consolidative transplantation following CAR-T cell-mediated remission remains controversial. We conducted a retrospective study to explore whether bridging to unrelated cord blood transplantation (UCBT) could improve the prognosis of patients entering remission after CAR-T therapy with different characteristics through subgroup analyses.

**Methods:**

We reviewed 53 patients with relapsed/refractory (R/R) B-cell acute lymphoblastic leukemia (B-ALL) successfully infused with CD19 CAR-T cells and achieved complete remission (CR). In this study, 25 patients received consolidative UCBT (UCBT group) and 28 patients did not accept any intervention until relapse (non-UCBT group). Subgroup analysis on prognosis was then performed according to gender, age, number of previous relapses, tumor burden, presence of poor prognostic markers, and structure of CAR.

**Results:**

Compared with the non-UCBT group, patients who underwent consolidative UCBT had better median event-free survival (EFS; 12.3 months vs. 6.2 months; P = 0.035) and relapse-free survival (RFS; 22.3 months vs. 7.2 months; P = 0.046), while no significant difference was found in overall survival (OS; 30.8 months vs. 15.3 months; P = 0.118). Subsequent multivariate analysis revealed that bridging to UCBT was a protective factor for RFS (P = 0.048) but had no significant effect on EFS (P = 0.205) or OS (P = 0.541). In the subgroup analysis, UCBT has an added benefit in patients with specific characteristics. Patients who experienced ≥2 relapses or with sustained non-remission (NR) showed better RFS (P = 0.025) after UCBT. Better EFS was seen in patients with poor prognostic markers (P = 0.027). In the subgroup with pre-infusion minimal residual disease (MRD) ≥5% or with extramedullary disease (EMD), UCBT significantly prolonged EFS (P = 0.009), RFS (P = 0.017), and OS (P = 0.026). Patients with occurrence of acute graft-versus-host disease (aGVHD) appeared to have a longer duration of remission (P = 0.007).

**Conclusion:**

Consolidative UCBT can, to some extent, improve clinical outcomes of patients with R/R B-ALL entering remission following CD19 CAR-T therapy, especially in patients with more recurrences before treatment, patients with poor prognostic markers, and patients with a higher tumor burden. The occurrence of aGVHD after UCBT was associated with better RFS.

## 1 Introduction

Chimeric antigen receptor (CAR)-T cell therapy has been proven to have remarkable efficacy in hematological malignancies in recent years and is considered one of the most promising targeted therapies for tumors. CD19-targeted CAR-T cell therapy has led to a paradigm shift in the treatment of relapsed and refractory (R/R) B-cell acute lymphoblastic leukemia (B-ALL). Numerous clinical trials revealed that patients treated with CD19 CAR-T cells can achieve a complete remission (CR) rate of 75%–93%. Despite the impressive results, however, approximately 38%–61% of patients eventually relapsed during long-term follow-up (1–8). Researchers have proposed a series of solutions to reduce the recurrence rate after CAR-T cell therapy such as optimizing CAR structure (7, 8), designing artificial antigen-presenting cells (AAPCs) (9), binding immunological checkpoint inhibitors (10), sequentially administering two groups of CAR-T cells (11), and bridging to transplantation. Some studies speculated that the combination of CAR-T therapy and transplantation prolonged survival and provided patients with more opportunities (12–14).

However, the role of consolidative transplantation following CAR-T therapy remains controversial while the effect can be influenced by pretreatment patient characteristics, lymphodepletion regimen, structure of CAR, and post-CAR-T therapy parameters (12). In some groups, it increases the economic burden and risk of death related to transplantation. It is necessary to weigh the advantages and disadvantages.

Equally important, which source of stem cells for transplantation to choose is inconclusive. Current studies emphasize the interface between allogeneic hematopoietic stem cell transplantation (allo-HSCT) and CAR-T cell therapy, as allo-HSCT is considered the only recognized curative cellular therapy for patients with B-ALL. Few studies have focused on unrelated cord blood transplantation (UCBT) following CAR-T therapy. Umbilical cord blood (UCB) is gradually being considered as an alternative source of peripheral blood progenitor cells (PBPCs) or bone marrow (BM) transplantation, particularly when a human leukocyte antigen (HLA)-matched donor is not available. A retrospective study determined the optimal role of UCBT in adults with acute leukemia, and showed that leukemia-free survival (LFS) in patients after UCBT was equivalent to that after PBPC or BM transplantation (15). Thus, UCBT may also be considered as an option to improve the prognosis of patients who have undergone CAR-T therapy. Another study conducted at our center showed that patients who received consolidative UCBT after CAR-T-induced remission had a 26.7% 2-year cumulative incidence of relapse (CIR) (16), so it is worth exploring if UCBT can benefit patients who have obtained remission after CAR-T therapy. Our research aims to explore the role of consolidative UCBT in patients receiving CD19 CAR-T cells and which characteristics of patients can influence the effect of UCBT more obviously.

## 2 Materials and Methods

### 2.1 Patients

From January 2016 to April 2021, we reviewed patients with R/R B-ALL who were successfully infused with CD19 CAR-T cells at the First Affiliated Hospital of USCT (Anhui Provincial Hospital) and the Second Hospital of Anhui Medical University (SHAMU). The medical ethics committee of the two hospitals reviewed and approved the study protocols. Fifty-three patients were consecutively enrolled in this non-randomized clinical study according to exclusion criteria including the following: 1) unable to evaluate the effectiveness of CAR-T cell therapy, (2) failure to achieve CR after CAR-T cell therapy, (3) contraindications in terms of critical organ insufficiency and uncontrollable infections, and (4) a history of transplantation before CAR-T therapy. Twenty-five patients received consolidative UCBT at our center selectively, depending on pre-UCBT assessment including disease features, treatment history, comorbidities, and personal reasons including patient preference and economic considerations. In conformity with the Declaration of Helsinki, informed consent was provided from each participant.

### 2.2 Procedures

#### 2.2.1 Chimeric Antigen Receptor-T Cell Therapy Protocol

All included patients were treated with conditioning chemotherapy including fludarabine (FLU, 30 mg/m^2^ × 3 days) in combination with cyclophosphamide (CY, 300 mg/m^2^ × 3 days) before the intravenous infusion of cryopreserved CD19 CAR-T cells at a dose of 1 × 10^6^ cells/kg body weight. The CAR in this study consists of an CD19-specific single-chain antibody fragment (scFv) derived from FMC63 fused to a modified IgG4-hinge spacer, a costimulatory molecule including CD28 alone or both CD28 and CD137 (4-1BB), and a CD3ζ signaling domain. Enrichment of CAR-T cells from patient’s PBMCs was done using CD4-magnetic beads (Miltenyi Biotec GmbH) and CD8-magnetic beads (Miltenyi Biotec GmbH).

#### 2.2.2 Unrelated Cord Blood Transplantation Procedures

The selection of cord blood and HLA typing have been previously described (17). Concisely, molecular techniques with minimum antigenic segmentation level resolution for HLA-A and HLA-B and allele-level resolution for DRB1 were used when performing HLA typing. All recipients obtained single-unit cord blood from the Chinese Cord Blood Bank. Each unit of cord blood was high-resolution matched to the recipient’s HLA-A and HLA-B antigens and HLA-DRB1 and had a minimum of 3.0 × 10^7^ total nucleated cells (TNCs)/kg of body weight and 1.2 × 10^5^ CD34^+^ cells/kg of body weight until frozen. All recipients were given myeloablative regimen including FLU/BU/CY (FLU, 30 mg/m^2^ per day for 4 days; busulfan, total 12.8 mg/kg, 0.8 mg/kg every 6 h for 4 days; CY, 60 mg/kg daily for 2 days), FLU/BU/CY plus carmustine (BCNU) (250 mg/m^2^), FLU/CY plus total body irradiation (TBI; total 12 Gy, 4 fractions) or FLU/BU/CY plus semustine (320 mg/m^2^). A co-application of mycophenolate mofetil and cyclosporin A was used as prophylaxis for graft-versus-host disease (GVHD).

### 2.3 Definitions

Bone marrow morphology with ≤5% blasts, no evidence of circulating blasts, and no extramedullary infiltration were considered as CR. Minimal residual disease (MRD)-negative CR was characterized as no immunophenotypically abnormal blasts detected in peripheral blood (PB)/BM by multiparametric flow cytometry (FCM). MRD status was assessed by 10-color FCM with a sensitivity of 10^4^ nucleated cells. Disease relapse was defined as ≥5% of blasts in BM, reappearance of blasts in the PB, or extramedullary infiltration after CR. The endpoints were event-free survival (EFS), relapse-free survival (RFS), and overall survival (OS). We calculated EFS as the time interval from CAR-T cell infusion to disease progression (including MRD-positive and gene recurrence), relapse, or death, whichever came first, or last visit. RFS was calculated from the date of CAR-T cell infusion to the date of relapse, death, or last visit. For OS, death as the final endpoint was caused by any factor or the final follow-up date could be used. Acute GVHD (aGVHD) was assessed according to the Mount Sinai Acute GvHD International Consortium (MAGIC) criterion (18), and chronic GVHD (cGVHD) was assessed according to the consensus criteria of the National Institutes of Health (19).

### 2.4 Statistical Analysis

Descriptive statistics were used to present the characteristics of the patients. The difference in non-relapse mortality (NRM) rate between two groups was determined by Fisher’s exact test. Kaplan–Meier analysis and Cox regression model were applied to perform univariate and multivariate analysis on factors affecting the survival of overall patients, respectively. The median EFS, RFS, and OS were demonstrated by Kaplan–Meier curves and were compared by log-rank test. The hazard ratio (HR) and 95% CI for EFS, PFS, and OS for subgroup analysis were estimated using a stratified Cox regression model and visualized by a forest plot. A two-tailed P value <0.05 was considered statistically significant. All statistical analyses were conducted using Statistical Product and Service Solutions (SPSS) 26.0, and GraphPad Prism 8.00 software (GraphPad Software, La Jolla, CA, USA) was used to create figures.

## 3 Results

### 3.1 Patients

A total of 53 patients with B-ALL treated with CD19 CAR-T cells were consecutively enrolled. Characteristics of patients with a median age of 28 (range 3–66 years) years are presented in **Table 1**. Twenty-two (41.5%) patients experienced less than 2 relapses, and 31 (58.5%) patients experienced ≥2 relapses or remain in non-remission (NR). Thirty-six (67.9%) patients had poor prognostic markers including *TP53*, *BCR-ABL1*, *E2A-PBX1*, and *MLL-AF4* and complex karyotype before CAR-T therapy. Furthermore, 43 (81.1%) patients had MRD >5% or with extramedullary disease (EMD) before CAR-T treatment, and 10 (18.9%) patients had MRD <5%. Thirty-eight (71.7%) patients were infused with CAR-T cells incorporating a CD28 co-stimulation domain, and 15 (28.3%) patients with CAR-T cells contained both CD28 and 4-1BB. All patients achieved CR before transplant, while 3 patients remained MRD-positive.

**Table 1:** Characteristics of the 53 patients.

Characteristics	Overall number (%)
**Bridging to UCBT**	
Yes	25 (47.2)
No	28 (52.8)
**Age (years)**	28 (3-66)
**Gender**	
Men	20 (37.7)
Women	33 (62.3)
**Number of relapses**	
<2	22 (41.5)
≥2 or NR	31 (58.5)
Poor prognostic markers*	36 (67.9)
**Previous EMD**	12 (22.6)
**Pre-infusion tumor burden**	
MRD ≥5% or with EMD	43 (81.1)
MRD <5%	10 (18.9)
**CAR structure**	
CD28	38 (71.7)
CD28/4-1BB	15 (28.3)
**Pre-transplant BM-MRD**	
MRD negative CR	22 (88.0)
MRD positive CR	3 (12.0)
**Transplant-related complications**	
aGVHD	11 (44.0)
cGVHD	1 (4.0)
PES	19 (76.0)
TRM	4 (16.0)

Data are presented as the median (range) or count (percentage).

UCBT, unrelated cord blood transplantation; EMD, extramedullary disease; MRD, minimal residual disease; CAR, chimeric antigen receptor; BM, bone marrow; CR, complete remission; aGVHD, acute graft-versus-host disease; cGVHD, chronic graft-versus-host disease; PES, pre-engraftment syndrome; TRM, transplantation-related mortality.

^*^Include: Complex karyotype, BCR-ABL1, MLL-AF4, TP53, E2A-PBX1.

Twenty-five patients received UCBT after CAR-T cell therapy (the UCBT group); 28 patients did not accept any treatment until relapse (the non-UCBT group). The median interval from CAR-T therapy to UCBT was 66 (range 30–268 days) days; 17 patients received UCBT within 3 months following CAR-T therapy. The median infused TNC count and CD34^+^ cells were 2.79 (range, 1.24–9.80) × 10^7^/kg and 1.97 (range, 0.69–7.15) × 10^5^/kg, respectively. Among the patients who underwent UCBT after CAR-T therapy, 11 (44.0%) patients had aGVHD and one patient had cGVHD in the follow-up. Pre-engraftment syndrome (PES) was observed in 19 (76.0%) patients (**Table 1**).

### 3.2 Overall Outcomes

**Figure 1:**
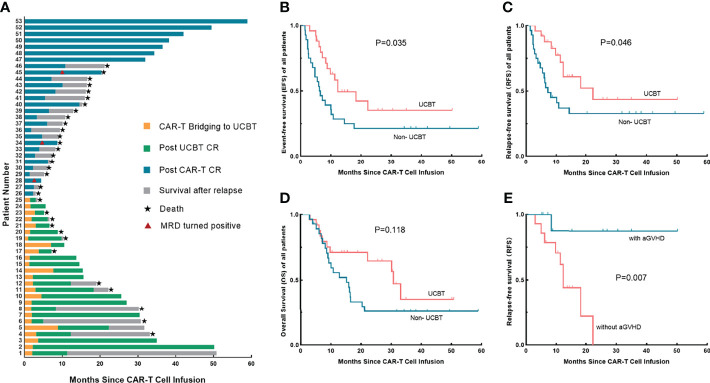
Treatment responses and long-term survival of each patient, survival analysis between the UCBT group and the non-UCBT group, and the association between aGVHD and RFS in the UCBT group. **(A)** Disease status from CAR-T cell infusion to the end of follow-up of each patient. **(B–D)** Differences of EFS **(B)**, RFS **(C),** and OS **(D)** between the UCBT group and the non-UCBT group in overall patients. **(E)** The association between the occurrence of aGVHD and RFS of patients underwent consolidative UCBT after entering remission by CAR-T therapy. UCBT, unrelated cord blood transplantation; aGVHD, acute graft-versus-host disease; EFS, event-free survival; RFS, relapse-free survival; OS, overall survival; CAR, chimeric antigen receptor.

Disease status of 53 patients from the day of CAR-T cell infusion to the end of follow-up was shown in **Figure 1A**. The NRM rates in the UCBT and non-UCBT groups were 33.3% and 5%, respectively; no significant difference was found by Fisher’s exact test (P = 0.128) between the two groups. In the UCBT group, 10 (40.0%) patients relapsed at a median time of 6.2 months (range 1.4–22.3 months) including one CD19-negative relapse and one central nervous system leukemia (CNSL), and one patient underwent transformation from ALL to myelodysplastic syndrome (MDS) and eventually progressed to acute myeloid leukemia (AML). Ten (40.0%) patients remained disease-free until the end of follow-up; one patient was lost to follow-up at 5.6 months of remission. Four patients died because of transplant-related complications including GVHD, thrombotic microangiopathy (TMA), and severe infection. Among the patients who relapsed after UCBT, eight patients received reinfusions of CAR-T cells after relapse, five patients achieved CR again, and two of them remained in MRD-negative remission until the end of follow-up. The remaining two patients who received only chemotherapy or supportive therapy showed poor prognosis and died of primary disease in the short term. In the non-UCBT group, 18 (64.3%) patients experienced relapses at a median time of 6.5 months (range 1.4–58.9 months). Six patients achieved sustained MRD-negative remission without other interventions after CAR-T cell infusion until the end of follow up, one patient remained in MRD-negative remission and was lost to follow-up at 34.3 months, one patient showed a positive MRD at 36.5 months, and one patient showed a positive *P210* at 17.7 months.

### 3.3 Survival Analysis

Initially, we performed an analysis of the factors associated with overall prognosis (**Table 2**). Univariate analysis indicated a significant difference in survival between the two groups, the estimated median EFS (12.3 months vs. 6.2 months; P = 0.035) and RFS (22.3 months vs. 7.2 months; P = 0.046) were better in the UCBT group than those in the non-UCBT group, while the OS showed no difference (30.8 months vs. 15.3 months; P = 0.118) (**Figures 1B–D**). Subsequent multivariate analysis for factors with P < 0.2 in the univariate analysis showed that consolidative UCBT was an independent protective factor for RFS (P = 0.048), but with no significant effect on overall EFS (P = 0.205) or OS (P = 0.541). Multivariate analysis also showed a worse EFS (P = 0.069) and OS (P = 0.045) in patients with poor prognostic markers.

**Table 2:** Factors affecting EFS, RFS, and OS in overall patients.

Items	EFS			RFS			OS		
	Median/mean	Log-rank χ^2^	P	Median/mean	Log-rank χ^2^	P	Median/mean	Log-rank χ^2^	P
**Univariate analysis**									
Bridging to UCBT									
No	6.2	4.456	0.035*	7.2	3.990	0.046*	15.3	2.522	0.112
Yes	12.3			22.3			30.8		
CAR structure									
CD28	10.0	0.084	0.772	12.3	0.113	0.737	16.6	0.155	0.694
CD28/4-1BB	9.8			12.3			30.8		
Age (years)									
<25	18.3	3.362	0.067	22.3	1.198	0.274	33.2	3.090	0.079
≥25	7.2			10.1			15.3		
Poor prognostic markers*									
No	22.3	2.985	0.084	6.5	1.946	0.163	38.0	3.610	0.057
Yes	8.2			2.7			19.3		
Number of relapses									
<2	14.5	0.817	0.366	18.3	0.788	0.375	22.2	0.531	0.466
≥2 or NR	8.3			11.3			16.5		
Tumor burden									
MRD ≥5%/EMD	8.8	0.001	0.979	6.1	0.514	0.474	16.5	0.099	0.754
MRD <5%	10.8			4.1			16.6		
**Multivariate analysis**									
**Items**	**HR (95% CI)**		**P**	**HR (95% CI)**		**P**	**HR (95% CI)**		**P**
Bridging to UCBT	0.562 (0.232–1.370)		0.205	0.456 (0.210–0.992)		0.048*	0.737 (0.276–1.964)		0.541
Age (years)	1.293 (0.521–3.205)		0.580	1.865 (0.788–4.413)		0.156	1.679 (0.612–4.601)		0.314
Poor prognostic markers	2.033 (0.945–4.375)		0.069	–		–	2.411 (1.022–5.688)		0.045*

EFS, event-free survival; RFS, relapse-free survival; OS, overall survival; UCBT, unrelated cord blood transplantation; CAR, chimeric antigen receptor; NR, non-remission; MRD, minimal residual disease; EMD, extramedullary disease; HR, hazard ratio; CI, confidence interval.

^*^Include: Complex karyotype, BCR-ABL1, MLL-AF4, TP53, E2A-PBX1.

**Figure 2:**
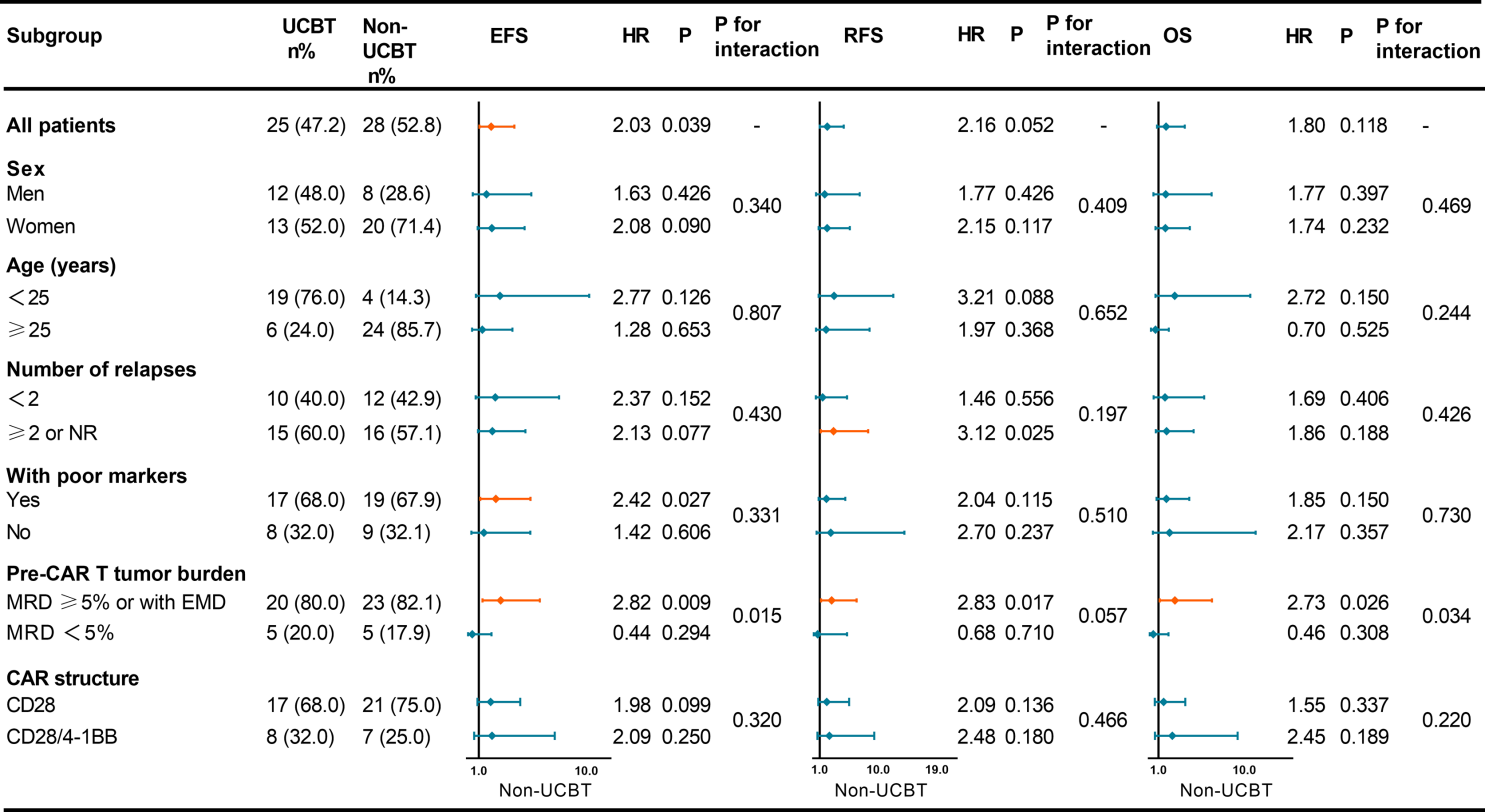
Forest plot showing the hazard ratios for EFS, RFS, and OS of various subgroups. UCBT, unrelated cord blood transplantation; EFS, event-free survival; RFS, relapse-free survival; OS, overall survival; CAR, chimeric antigen receptor; NR, non-remission; MRD, minimal residual disease; EMD, extramedullary disease; HR, hazard ratio.

Comparison of baseline characteristics of the two groups revealed no significant differences in gender, number of relapses, poor prognostic markers, pre-infusion tumor burden, and CAR structure; however, there were differences in the age distribution (**Table 3**). Considering if those factors may potentially associate with the influence of consolidative UCBT on the prognosis of patients, we subsequently subgrouped the patients to determine which group of patients were more likely to benefit from consolidative UCBT (**Figure 2**). In patients who experienced ≥2 relapses or sustained NR, UCBT prolonged RFS (P = 0.025) compared to non-UCBT. Consolidative UCBT had significant influences in EFS (P = 0.027) of patients with poor prognostic markers including *MLL-AF4*, *BCR-ABL1*, *TP53*, *E2A-PBX1*, and complex karyotype, while no significant effect was shown in patients without those markers. In the subgroup with MRD ≥5% or with EMD before infusion, UCBT prolonged EFS (P = 0.009), RFS (P = 0.017), and OS (P = 0.026) independent of poor prognostic markers; the improvement was not observed in patients with pre-infusion MRD <5%. No significant effect of UCBT was seen in either group subdivided according to gender (men and women), CAR structure (CD28 and CD28/4-1BB), and age (<25 years and ≥25 years).

**Table 3:** Patient characteristics among the two groups.

Characteristics	Non-UCBT group (N = 27)	UCBT group (N = 29)	P value (Non-UCBT vs. UCBT)
Age (years)			<0.001**
<25	4 (14.3)	19 (76.0)	
≥25	24 (85.7)	6 (24.0)	
Gender			0.145
Men	8 (28.6)	12 (48.0)	
Women	20 (71.4)	13 (52.0)	
Number of relapses			0.833
<2	12 (42.9)	10 (40.0)	
≥2 or NR	16 (57.1)	15 (60.0)	
Poor prognostic markers*			0.991
No	9 (32.1)	8 (32.0)	
Yes	19 (67.9)	17 (68.0)	
Pre-infusion tumor burden			0.842
MRD <5%	5 (17.9)	5 (20.0)	
MRD ≥5% or with EMD	23 (82.1)	20 (80.0)	
CAR structure			0.572
CD28	21 (75.0)	17 (68.0)	
CD28/4-1BB	7 (25.0)	8 (32.0)	

Data are presented as count (percentage).

*P < 0.05 (bilateral); **P < 0.01 (bilateral).

UCBT, unrelated cord blood transplantation; NR, non-remission; MRD, minimal residual disease; EMD, extramedullary disease; CAR, chimeric antigen receptor.

^*^Include: Complex karyotype, BCR-ABL1, MLL-AF4, TP53, E2A-PBX1.

In our study, time between CAR-T cell infusion and UCBT did not influence EFS (P = 0.360), RFS (P = 0.413), or OS (P = 0.204) of the patients. Four patients bridging to UCBT at 3 months after CAR-T therapy still showed sustained remission. Our further exploration suggested a correlation between aGVHD after UCBT and prognosis, which showed that patients who had aGVHD tend to have a better RFS (P = 0.007) (**Figure 1E**), although EFS (P = 0.113) and OS (P = 0.593) did not significantly prolong, and the results need to be further confirmed by a large sample.

## 4 Discussion

While CAR-T cell therapy increases the remission rate of patients diagnosed with R/R B-cell hematological malignancies, the limited duration of remission and unsatisfactory long-term survival present challenges for CAR-T cell therapy. The two relapse patterns after CAR-T therapy include CD19-positive relapse and CD19-negative relapse. CD19-positive relapses are commonly associated with decreased CAR-T cell persistence, low potency and poor response of CAR, and transient B-cell aplasia (20). The mechanism of this kind of relapse may be related to CD19 gene mutation leading to CD19 loss (21), selection and lineage switch by immune pressure (22, 23), and trogocytosis and cooperative killing (24). Prevention for relapse after CAR-T therapy includes improving the structure of CAR (7, 25), optimizing lymphodepletion regimen (26), dual/multi-targeted CAR-T cells (23, 27), sequential infusion of two groups of CAR-T cells (11, 28), CAR-T cell combined with immune checkpoint inhibitor (10), and consolidative transplantation after CAR-T therapy (12–14, 29–33). HSCT has been chosen in several centers to consolidate remission after CAR-T treatment. Clinical studies by Lee et al. (14) demonstrated a decline in relapse rate in pediatric patients who were consolidated with HSCT. Hay et al. (34) reported a phase I/II data investigating the role of 4-1BB CD19 CAR-T cells in adult ALL, demonstrating prolonged EFS in patients who received HSCT after CAR-T therapy. A clinical study by Shalabi et al. (29) showed that CAR-T cell therapy combined with HSCT could synergistically improve LFS. However, whether to consolidate with transplantation after CAR-T is still a critical question, since treatment-related morbidity and mortality need to be balanced against the risk of relapse. Functional CAR-T cells that persisted in patients may be destroyed by transplantation and losing their antitumor activity. And there are some studies showing that no difference in prognosis was seen in patients who did and did not receive consolidative transplantation after fusion of CD28-based CD19 CAR-T therapy (6, 35).

Another issue to be addressed is which source of transplantation should be chosen. Current studies have focused on consolidative HSCT after CAR-T therapy, with fewer studies on UCBT. Stem cells can be obtained from BM, PB, or UCB. UCB offers benefits such as rapid acquisition, less constricted HLA matching, and lower rates of GVHD (36–38). A comparative analysis of UCB and BM in children with acute leukemia demonstrated that 4-6/6 HLA-matched UCB provided a similar probability of LFS to matched BM (39). Thus, we assumed that UCBT may serve as an alternative for patients without a compatible donor. However, there is still a deficiency of data on the outcomes of CAR-T therapy bridging to UCBT. Another study in our center showed a relatively high 2-year CIR in patients who received consolidative UCBT following CAR-T-mediated remission (16), so it is worth exploring whether UCBT can benefit patients who achieved remission after CAR-T therapy or not.

In this retrospective study, 25 of the 53 patients received subsequent UCBT after CAR-T therapy, and the other 28 received CAR-T therapy alone. In overall patients, the NRM rate appeared to be higher in the UCBT group, but there was no statistical difference between the two groups, and UCBT contributed a significant improvement in RFS, while there was no significant effect on EFS and OS. In studies focusing on consolidative allo-HSCT after CAR-T, the effect of HSCT was influenced by many potential factors including complex karyotypes, certain genes associated with poor prognosis, leukemia burden, number of relapses, high lactate dehydrogenase (LDH) levels, lymphodepletion regimen, and constructure of CARs (12). Therefore, we conducted a subgroup analysis to explore factors influencing the effect of UCBT.

A high pre-infusion tumor burden had been reported to increase the relapse rate after CAR-T therapy (6, 13, 40). A clinical trial showed that consolidative HSCT significantly prolonged EFS and RFS in patients with a high leukemia burden (13). In our study, we observed a pronounced influence of consolidative UCBT on patients with MRD ≥5% or with EMD, suggesting that patients with a higher tumor burden would be more likely to benefit from consolidative UCBT. Some studies have found that surface CAR expression was inversely correlated with tumor burden due to receptor internalization (41–43); this may contribute to a higher probability of relapse in patients with a high tumor burden. Those findings demonstrate the necessity of consolidative transplantation for patients with a high tumor burden after CAR-T therapy. In addition, we found that the number of relapses can influence the effect of UCBT, patients who experienced fewer than 2 relapses did not benefit from consolidative UCBT, while in patients with recurrent relapses or sustainable NR, consolidative UCBT improved their RFS significantly. As for patients with poor prognosis markers, while worse overall EFS and OS were observed in multivariate analysis, those patients showed an improved EFS after bridging to UCBT. *MLL/AF4*, *BCR/ABL1*, *TP53*, and *E2A/PBX1* complex karyotypes are reported to indicate an inferior prognosis in B-ALL patients (44–48). Consolidative UCBT after CAR-T therapy may achieve a longer molecular response.

According to criteria published by Gökbuget et al. (49) age is the most important prognostic factor for ALL, and the prognosis of patients deteriorates with increasing age. Apart from that, some studies showed that adult patients are more likely to benefit from consolidative allo-HSCT, while younger patients can achieve durable remission without a consolidating allo-HSCT (4, 8). As the younger patients took a larger proportion in the UCBT group in our study, we further grouped the patients according to age (<25 years vs. ≥25 years) to reduce the influence of the confounding variable; the results showed that consolidative UCBT did not affect survival of patients in either group.

Another factor that can affect prognosis is the co-stimulatory structural domain of CAR. Preclinical studies indicated a relatively short duration after infusion of CAR-T cells with CD28 co-stimulatory domain (50, 51); moreover, numerous investigations have revealed that 4-1BB-based CAR-T cells exhibited more durable persistence than CAR-T cells that contained CD28 co-stimulatory domain (20, 32, 33, 52, 53). However, the outcomes of CAR-T cell containing CD28 co-stimulatory domains varied significantly among different studies. A retrospective analysis showed no improvement in EFS or OS in patients using CD28-based CD19 CAR-T cells bridged with HSCT (6). A study in pediatric patients using CD28 co-stimulatory CD19 CAR-T cells (54) suggested that HSCT leads to better EFS. Several reports of treating patients with 4-1BB-based CD19 CAR-T cells (13, 34) exhibited better RFS with consolidative HSCT, whereas patients receiving 4-1BB-containing CD19 CAR-T cells in the global ELIANA trial show no benefit in OS (8). It is unscrupulous to conclude the influence of different CAR structures on the effect of post-CAR-T transplantation. In our study, we did not observe an improvement in prognosis by consolidative UCBT in patients receiving CD28-based CAR nor in those receiving CD28/4-1BB-based CAR. Considering the limited sample size and confounding factors, a comparative study is needed.

Our study demonstrated that consolidative UCBT after CAR-T therapy can improve the clinical outcomes in specific groups of patients; however, the recurrence rate after transplantation was relatively high (40.0%, 10/25). Patients without occurrence of aGVHD appeared to have a shorter duration of remission. The occurrence of aGVHD after UCBT may indicate better RFS. A study showed that time from CAR-T cell application to transplantation associated with the risk of death (55). Some researchers recommended early consolidative UCBT to maximize the benefit, considering that approximately 10% of patients relapsed after CAR-T therapy within 3 months (12). However, our study did not show a correlation between the interval and long-term survival; among the 10 patients in the UCBT group who achieved sustained remission, four patients had an interval ≥3 months from CAR-T infusion to transplantation. Thus, the optimal time for consolidative transplantation needs to be determined by multicenter and large-sample studies. It is imperative to seek a treatment option for patients who relapse after transplantation because these patients generally have a poor prognosis. Eight patients accepted re-infusion of CAR-T cells in this study, five of them achieved CR and the median OS after relapse was 21.9 months (range 9.4–39.5 months), the remaining two patients who did not receive a second CAR-T therapy died rapidly after relapse. Comparative studies with larger samples are necessary to explore the role of re-infusion of CAR-T cells.

Our study has its shortcomings due to its retrospective nature and the uniformity in baseline characteristics of patients; election bias on transplantation may also exist. However, the results from real-world data by subgroup analysis are still informative. Large-sample studies with consistent patient baseline characteristics are still needed.

## 5 Conclusion

To conclude, our study showed that consolidative UCBT provides an option for post-CAR-T consolidation. In patients with a history of ≥2 relapses or sustained NR, with poor prognostic markers, or with MRD ≥5% or EMD, long-term survival was significantly improved by consolidative UCBT. In contrast, UCBT does not necessarily result in a better prognosis for patients with a lower number of recurrences, no prognostic markers, and a lower tumor burden. For posttransplantation relapse, re-infusion of CAR-T cells may be an option to consider.

## Data Availability Statement

The original contributions presented in the study are included in the article/supplementary material. Further inquiries can be directed to the corresponding author.

## Ethics Statement

This study was approved by the medical ethics committee of the First Affiliated Hospital of USTC (Anhui Provincial Hospital), Hefei, China (2016-101), and the Second Hospital of Anhui Medical University (SHAMU). All patients gave their written informed consent in accordance with the Declaration of Helsinki.

## Author Contributions

QX, LX, and XBW analyzed the data and wrote the paper. FA, HX, LW, LG, XHZ, KS, WY, XW, JT, HL, XL, XYZ, ZZ, ZS, and XBW provided the study patients. XBW designed the research and edited the article. All authors contributed to the article and approved the submitted version.

## Funding

This study received funding from the National Natural Science Foundation of China #1 under Grant 82170221. The funder was not involved in the study design, collection, analysis, interpretation of data, the writing of this article or the decision to submit it for publication. All authors declare no other competing interests.

## Conflict of Interest

The authors declare that the research was conducted in the absence of any commercial or financial relationships that could be construed as a potential conflict of interest.

## Publisher’s Note

All claims expressed in this article are solely those of the authors and do not necessarily represent those of their affiliated organizations, or those of the publisher, the editors and the reviewers. Any product that may be evaluated in this article, or claim that may be made by its manufacturer, is not guaranteed or endorsed by the publisher.
